# Effect of Biomin F toothpaste and Diode laser on remineralization of white spot lesions (in vitro study)

**DOI:** 10.1186/s12903-024-04589-9

**Published:** 2024-07-30

**Authors:** Amira I. Eldeeb, Nazla O. Tamish, Ahmed M. Madian

**Affiliations:** Department of Orthodontics, Faculty of Dentistry, Alexandria University, Alexandria, Egypt

**Keywords:** Biomin, Laser therapy, WSL, Tooth remineralization

## Abstract

**Background:**

White-spot lesions are considered an initial carious stage characterized by an outer enamel layer with significantly reduced mineralization. This study was conducted to assess the combined effect of Biomin F toothpaste and Diode laser on remineralization of white spot lesions.

**Materials and methods:**

An invitro study conducted on a total of 30 premolars divided into three groups; Group A (Biomin F Tooth paste), Group B (Biomin F with laser application for 30 sec), Group C (Negative control). The three groups were submitted to three stages; stage 1:Baseline,stage 2:After demineralization ,and stage 3:After remineralization. In each stage, elemental analysis(calcium, phosphorus, and fluoride)was measured quantitatively using Energy Dispersive X-ray (EDX) analysis and qualitatively by micrographs using scanning electron microscope. The data were tested to find significant difference between mineral changes during stages by using (ANOVA) test and Bonferroni test.

**Results:**

Calcium, phosphorus and fluoride ions decreased in all groups after demineralization. In stage 3, after application of remineralizing agents, Calcium ions increased significantly in groups A and B where *p*<.05. As regards to the phosphorus ions, a significant increase was observed in all groups with group A showed the highest gain as phosphorus level percentage change (%mass) was 56.52±18.02 . Fluoride ions increased significantly in groups A and B (*p*<0.05) but decreased significantly in group C. There was no statistical significant difference between group A and B (*p* ≥.05) in calcium, phosphorus, and fluoride level after remineralization.

**Conclusion:**

Within the limitation of the present study, we concluded that Biomin F toothpaste is promising in the repairing of white spot lesions on the surface of the demineralized enamel. Diode laser did not affect the remineralizing ability of Biomin F toothpaste.

## Introduction

Typically, patients seek orthodontic intervention to enhance their aesthetic appeal. Nonetheless, the use of fixed orthodontic devices could result in the development of white spot lesions (WSLs), posing an additional aesthetic challenge for the patient. Consequently, both the patient and orthodontist may experience disappointment upon the removal of the appliances [1].

White spot lesions are recognized as the initial stage of tooth decay, characterized by a surface enamel layer showing a significant reduction in mineral content [2]. This condition is prone to deteriorate, potentially necessitating invasive treatment [3]. Enamel demineralization and the development of WSLs advance swiftly [4], often developing within a few weeks [5].

Preventive approaches for WSLs during orthodontic treatment primarily involve the utilization of fluoride-releasing varnishes [6, 7], bonding materials, and cements [8, 9]. Additionally, the application of concentrated fluoride gels [10] and daily rinsing with mouthwash [11–14] are employed to reduce enamel demineralization.

While fluoride application is effective in halting WSLs, it comes with certain constraints. The consistent use of toothpaste containing fluoride necessitates a significant presence of bioavailable calcium and phosphate ions alongside fluoride ions [15]. Furthermore, fluoride exhibits diminished effectiveness when pH level drops below 4.5, a situation often instigated by bacterial actions [16].

When treating visible white spot lesions using concentrated fluoride agents (hypermineralization), the lesion is arrested at the surface instead of allowing saliva to promote remineralization in the deeper areas [16]. Presently, there are numerous methods available to halt or reverse the advancement of WSLs utilizing low levels of fluoride, such as casein phosphopeptides–amorphous calcium phosphate (CPP-ACP) [17], Nano-hydroxyapatite, Trimetaphosphate ion and Bioactive glasses [16, 18].

In recent times, bioactive glasses have emerged as a notable advancement in dental applications, extensively researched in various studies targeting the treatment of white spot lesions through remineralization. These materials have the potential to rejuvenate and regenerate dental tissues by triggering apatite formation upon exposure to saliva or any other physiological fluid [16, 19]. These apatites can either be hydroxyapatites or fluorapatites, depending on whether fluoride is integrated into the glass structure chemical composition. Glasses contain fluoride exhibit "smart" properties, demonstrating remineralization activity enhancement in low pH environments. Biomin F is recognized as a bioactive glass-based toothpaste that incorporates low concentration of fluoride (~600ppm) to aid in the remineralization of enamel.

Biomin F possesses the characteristic of prolonged fluoride delivery over a 12-hour period through gradual dissolution of the glass [16]. This characteristic is due to the polymer that enhances the bond between the calcium in the bioglass material and the calcium on the enamel. This bonding reduces the leaching of bioactive glass material [20]. Biomin F contains small bioglass particles that aid in the infiltration of remineralizing agents into subsurface lesions [21, 22]. With its high phosphate content, Biomin F facilitates rapid apatite formation (within 6 hours) and contains fewer carbon impurities, thereby rendering enamel less soluble in acid [21, 22].

Laser technology has been utilized to decrease the rate of subsurface demineralization of enamel by modifying its crystalline structure, acid solubility, and permeability. However, it is crucial to apply lasers at a low energy level to maintain enamel integrity [23].

Following laser irradiation, enamel undergoes chemical and structural changes, including reduction in carbonates fusion and re-crystallization of hydroxyapatite crystals. These alterations enhance enamel's resistance against the acid attacks. Moreover, studies have demonstrated synergistic effects between laser treatment and topical fluoride application, leading to a significant reduction in the rate of enamel decalcification [18, 21].

Hence, this study was conducted for the evaluation of the combined impact of Biomin F toothpaste and diode laser on the remineralization of WSLs.

The null hypothesis of this study assumed that no significant difference is expected between the effect of Biomin F toothpaste coupled with diode laser and Biomin F alone on remineralization of white spot lesions.

## Materials and methods

This in-vitro study was conducted at the Department of Orthodontics, Faculty of Dentistry, Alexandria University and Scanning Electron Microscope unit, Faculty of Sciences, Alexandria University. The present study was approved by the Research Ethics Committee of the Faculty of Dentistry, Alexandria University (IRB:00010556– IORG:0008839).

### Sample size calculation

The sample size estimate was calculated based on an invitro study by Aidaros et al. (2022) [15] that aimed to evaluate and compare the remineralizing potential of dentifrices containing fluoride and bioactive glass on enamel by assessing the enamel structure and elemental analysis through Energy Dispersive X-ray Analysis (EDX). During sample size calculation, a beta error of up to 20% is accepted, with a study power of 80%. The alpha level was established at 5%, corresponding to a significance level of 95%. Statistical significance was assessed at a *p*-value < 0.05 [24]. The minimum required sample size was determined to be 9 teeth per group (number of groups=3) (Total sample size=27 teeth). Any withdrawal for any reason will be compensated by replacement to control for attrition (loss of specimen) bias. Therefore sample size will be increased to 10 teeth per group (number of groups=3) (Total sample size=30 teeth).

A total of 30 human premolars were collected from patients requiring premolars extraction during their orthodontic treatment in the Orthodontic Department, Faculty of Dentistry, Alexandria University, Egypt. Informed consent was obtained from all subjects and/or their legal guardian(s).

All patients were born and lived in areas where the typical concentration of fluoride in the drinking water was 0.36 mg/L [25]. Any calculus or tissue remnants were removed from the teeth using a scaler. Subsequently, the teeth were stored in saline until the commencement of the study.

**Fig. 1:**
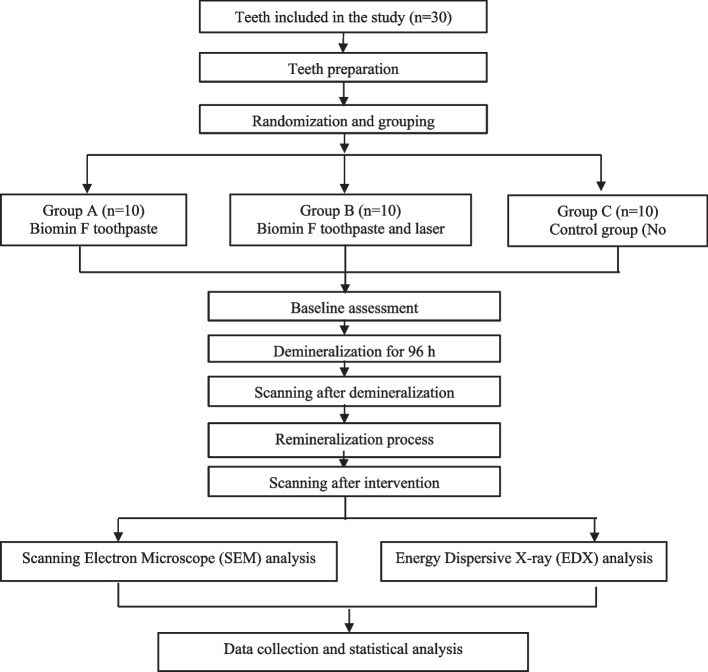
Flow chart showing procedure that has been done

Procedures for each group are shown in the flowchart (Fig. 1).

#### Collection of teeth

Thirty human premolars that were extracted for orthodontic purposes were chosen for inclusion in this study. All teeth were examined macroscopically using a magnifying loupe and fulfilled the following selection criteria: Intact buccal enamel surface, with no decalcifications, cracks, or stains.Teeth previously bonded or received any chemical treatment or decayed premolars were excluded from this study. The teeth were preserved in saline until the start of the experiment.

#### Teeth preparation

**Fig. 2:**
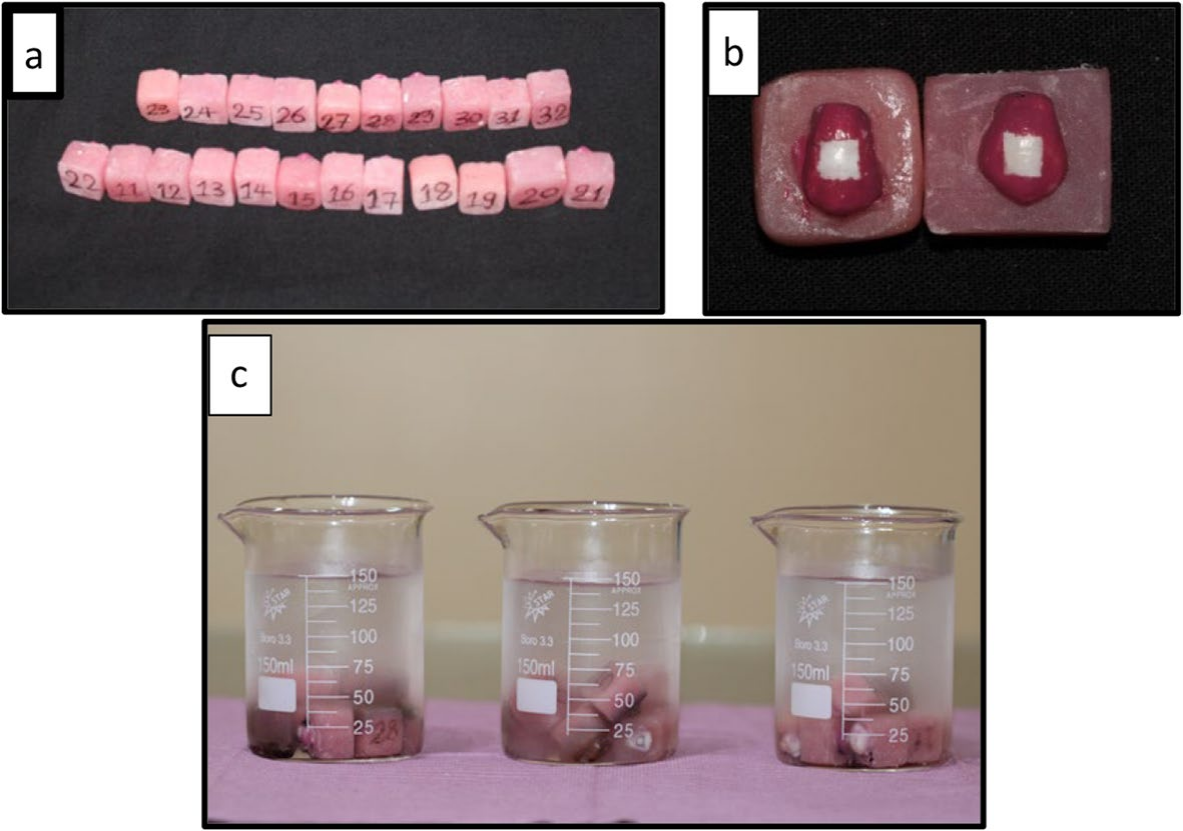
**a** Each tooth has a number typed at the base of acrylic block. **b** a window of 4x4mm in the middle third of the buccal surface of the tooth. **c** Each group was stored in a separate beaker containing artificial saliva and labeled with the group name

After recruiting the appropriate teeth, all remnants were removed and teeth were cleaned with fluoride free pumice and running water. The roots of the teeth were cut 2mm under cemento-enamel junction using a diamond disk under water cooling. The crowns were embedded in self cured acrylic resin and the buccal surfaces were directed upward for easy manipulation [15] (Fig. 2a). Each sample was covered with acid resistant varnish (nail polish) at all tooth crown surfaces, leaving a window of 4mm X4mm in the middle third of the buccal surface of the premolar (Fig. 2b).

### Randomization

Each tooth got a number from 1 to 30 typed at the base of the acrylic block using waterproof permanent marker. These numbers were used to randomly and equally assign the samples into 3 groups using computer generated random list.

#### Grouping of the teeth

Teeth were divided randomly into 3 groups

- Group A: Biomin F tooth paste.
- Group B: Biomin F with laser application for 30 sec .
- Group C: Negative control group (no treatment).

Each group was placed in its own labeled beaker which contained 150 mL of artificial saliva solution at room temperature of 37°C and neutral PH to replicate the oral environment (Fig. 2c). The materials used, their specifications, compositions and manufactures are present in (Table 1).

**Table 1:** The materials used and their specifications

**Solution**	**Composition**	**Usage**
Artificial saliva	• 2.20 g/L gastric mucin • 1.45 mmol/L CaCl2 2H2O • 5.42 mmol/L KH2PO4 • 6.50 mmol/L NaCl • 14.94 mmol/L KCl. PH was adjusted to 7.0 using KOH.	Storage medium for the samples.
Demineralizing solution	• 2.2 mM CaCl2 • 2.2 mM NaH2PO4 • 0.05 M lactic acid • 0.2 ppm fluoride. The pH was adjusted to 4.5 with 50% NaOH.	Artificial sub-surface lesion formation.
BioMinF Armour for teeth toothpaste (BioMin Technologies Ltd., London, UK)	• Fluoro Calcium-PhosphoSilicate (Biomin F) • Glycerin • Silica PEG 400 • Sodium-Lauryl Sulphate • Titanium Dioxide • Aroma • Carbomer • Potassium Acesulfame • Contains maximum 530ppm of available fluoride when packed.	Remineralizing agent

#### Intervention

The study was divided into a number of stages with various procedures as the following:

- First stage (Baseline) assessment

**Fig. 3:**
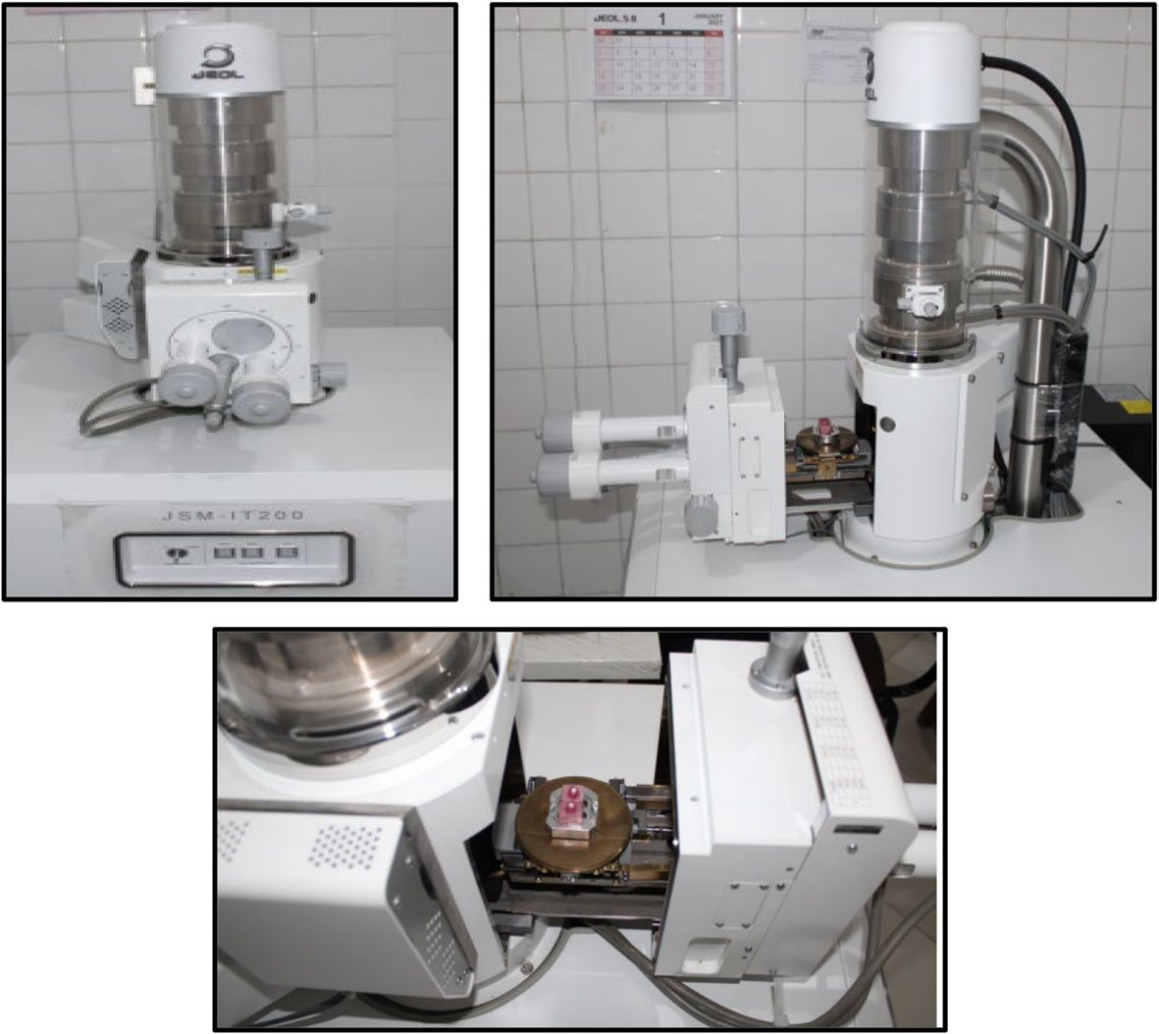
Scanning electron microscope attached with EDX unit

The assessment was conducted utilizing an environmental scanning electron microscope (JSM-IT 200-Japan) at Faculty of Sciences, Alexandria University. SEM attached with energy-dispersive X-ray (EDX) unit (Fig. 3).

The amount of remineralization was measured qualitatively by comparing the scanning electron microscope pictures and quantitatively by the Energy dispersive X-ray analysis that measured the average of three points selected in the area of concern. These values were taken at different stages of the study (before, after demineralization and after remineralization).

- Second stage (Demineralization process)

**Fig. 4:**
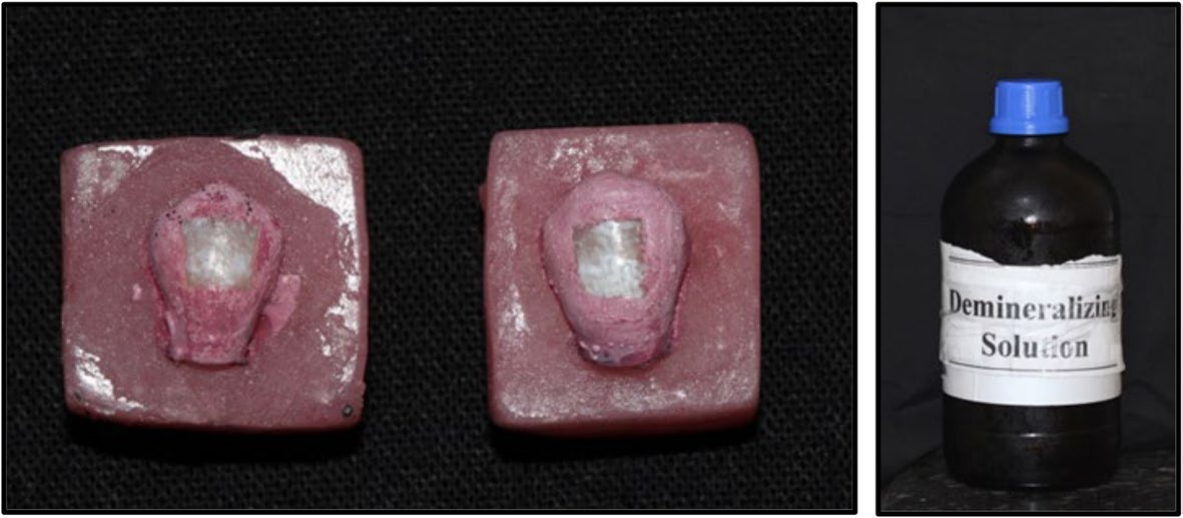
White spot lesions obtained after exposure to demineralizing solution

The teeth were immersed in the demineralizing solution for 96 hours at 37°C until white spot lesions were obtained (Fig. 4 and Table 1). The samples were removed from the solution and rinsed with distilled water to stop the demineralization process and to remove any residuals of the solution [21, 26]. At this stage, evaluations were carried out using Scanning Electron Microscope (SEM) analysis alongside Energy Dispersive X-ray Analysis (EDX).

- Third stage (Remineralization process)

**Fig. 5:**
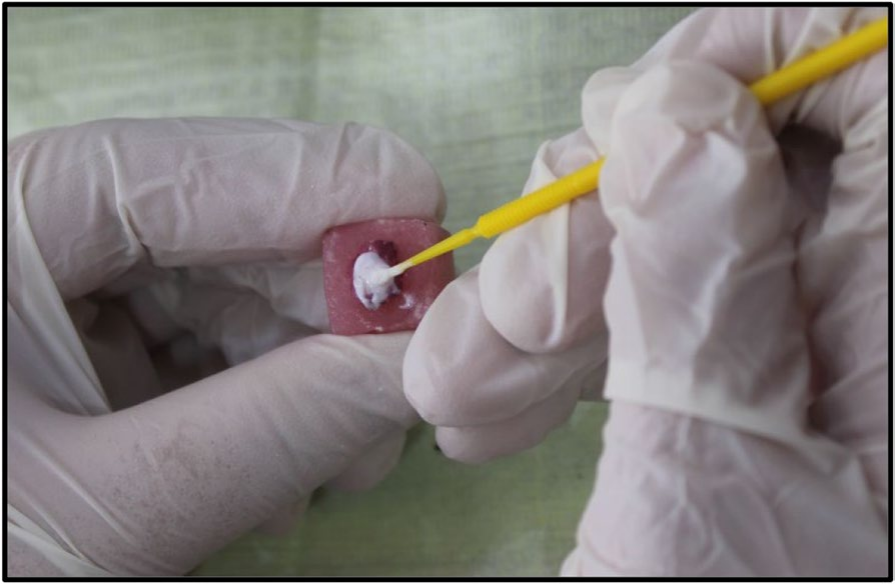
Biomin F toothpaste was applied in a circular motion

In group A: Biomin F toothpaste was applied in circular motion on the demineralized region using microbrush twice daily, each lasted for two minutes and left undisturbed for 30 seconds (Fig. 5). Then the samples were rinsed carefully with distilled water to remove any excess paste. Then it was stored in artificial saliva to mimic oral environment. This procedure was repeated for two weeks (Table 1) [15, 21].

**Fig. 6:**
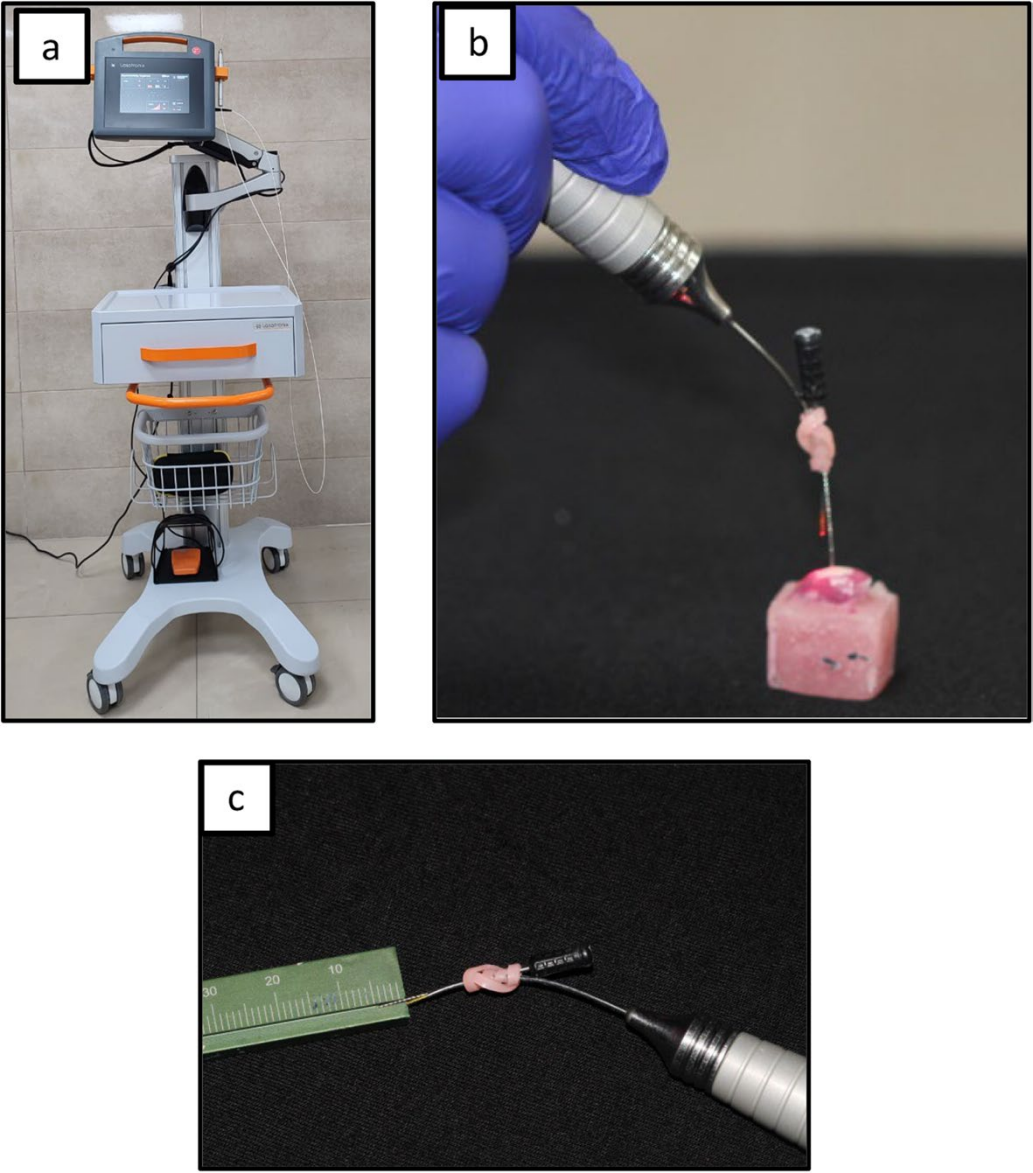
**a** Laser machine (Lasotronix-Boland), **b **and **c** showed The distance between the buccal surface of the tooth and laser fiber was kept at 5mm

In group B: The toothpaste was applied twice daily each for two minutes in a circular motion using a microbrush and left undisturbed for 30 seconds which lasted for two weeks, then laser was applied at the 14^th^ day (Fig. 6 and Table 2).

**Table 2:** Parameters of laser irradiation

Center wave lenghth(nm)	980
Operating mode	Continues wave(CW)
Power output(W)	0.5
Exposure time(s)	30
Radiant energy(J)	15
Optic conductor fiber diameter(μm)	320
Mode	Non contact mode

In group C: No treatment was received.

### Laser application

Laser irradiation (Lastronix-Boland) has been done on the 14^th^ day for 30 seconds after Laser beam activation with carbon particles and laser setting was done as shown in Table 2. The practitioner wore eye goggles for self protection from laser irradiation. The distance between the buccal surface of the tooth and laser fiber was kept 5mm using custom- made holder (Fig. 6 and Table 2) [15, 27].

The Scanning Electron Microscope assessment and Energy Dispersive X-ray Analysis (EDX) were repeated after remineralization process.

### Blinding

Technician of Scanning Electron Microscope attached with EDX unit and the statistician were blinded.

### Statistical analysis

- The data were gathered and inputted into the computer using the Statistical Package for the Social Sciences (SPSS) software program for statistical analysis (ver 25) [28].
- Kolmogorov-Smirnov test of normality revealed no significance in the distribution of the variables, so the parametric statistics were adopted [29].
- Data were described using minimum, maximum, mean, standard deviation, standard error of the mean, and 95% CI of the mean, 25^th^ to 75^th^ percentile [30].
- Comparisons were conducted among more than two independent normally distributed subgroups utilizing the one-way Analysis of Variance (ANOVA) test [31]. Post-hoc multiple comparisons [32] were performed using the Bonferroni method [33].
- Repeated measures analysis of variance was used [34]. Model assumptions were tested and found to be satisfactory with the exception of Mauchly’s test of sphericity [35], and when it was statistically significant denoting the violation of the assumption of sphericity, Greenhouse-Geisser correction was used [36]. Pair-wise comparison was done with Bonferroni correction.
- Linear trend analysis was used to test for within-subjects contrast [37].

Percentage change was calculated as follows:

$${\varvec{P}}{\varvec{e}}{\varvec{r}}{\varvec{c}}{\varvec{e}}{\varvec{n}}{\varvec{t}}{\varvec{a}}{\varvec{g}}{\varvec{e}}\boldsymbol{ }\;{\varvec{c}}{\varvec{h}}{\varvec{a}}{\varvec{n}}{\varvec{g}}{\varvec{e}}\boldsymbol{ }\left(\boldsymbol{\%}\right)=\frac{Measurement \left(after\right)-Measurement (before)}{Measurement (before)}\times 100$$

## Results

Tables 3, 4 and 5 shows the mean value (%mass) of mineral content (calcium –phosphate – fluoride) obtained from elemental analysis by using EDX for each tested group.

**Table 3:** Comparison of calcium level (% mass) at different repeated points of the measurement interval in the three studied groups

**Ca (% mass)**	**Progression**			Test of significance *p* value
	**Biomin** **(***n***=10)**	**Biomin and Laser** **(***n***=10)**	**Control** **(***n***=10)**	
**Baseline**				
- Min-Max	24.85-34.42	27.81-32.07	25.68-37.98	F_(df=2)_=0.651 *p*=.529 NS
- Mean±SD	31.34^**X,Z**^±2.74	29.98^**X,Z**^±1.40	30.91^**X**^±3.58	
- SE of Mean	0.87	0.44	1.13	
- 95.0% CI for Mean	29.38-33.30	28.98-30.98	28.35-33.47	
- 25^th^ Percentile – 75^th^ Percentile	30.35-33.17	29.13-31.51	28.99-33.42	
**After Demineralization**				
- Min-Max	18.95-32.08	16.38-24.42	16.07-23.84	F_(df=2)_=1.625 *p*=.216 NS
- Mean±SD	22.64^**Y**^±4.02	20.86^**Y**^±2.69	20.16^**Y,Z**^±2.59	
- SE of Mean	1.27	0.85	0.82	
- 95.0% CI for Mean	19.76-25.51	18.93-22.78	18.31-22.01	
- 25^th^ Percentile – 75^th^ Percentile	19.52-23.22	20.27-22.52	18.70-22.04	
**After Remineralization**				
- Min-Max	21.68-36.41	25.45-31.77	19.58-25.85	F_(df=2)_=16.936 *p*<.001*
- Mean±SD	29.49^**a,b,X,Z**^±3.99	29.14^**a,b,X,Z**^±2.39	22.66^**c,Y,Z**^±2.14	
- SE of Mean	1.26	0.76	0.68	
- 95.0% CI for Mean	26.64-32.35	27.43-30.85	21.13-24.19	
- 25^th^ Percentile – 75^th^ Percentile	28.18-31.48	26.51-30.97	21.18-25.07	
**One-way repeated measures analysis** *p* value	F_(GG)(df=1.260)_=16.179 *p*=.001*	F_(df=2)_=71.922 *p*<.001*	F_(GG)(df=1.186)_=44.448 *p*<.001*	
**Percentage Change (%) (Baseline vs After Demineralization)**				
- Min-Max	-41.15 - 3.45	-44.43 - -13.81	-57.69 - -19.24	F_(df=2)_=0.710 *p*=.501 NS
- Mean±SD	-27.56±12.49	-30.26±10.14	-33.77±12.26	
- SE of Mean	3.95	3.21	3.88	
- 95.0% CI for Mean	-36.50 - -18.63	-37.51 - -23.01	-42.54 - -25.00	
- 25^th^ Percentile – 75^th^ Percentile	-34.88 - -23.74	-36.79 - -21.20	-44.48 - -23.62	
**Percentage Change (%) (Baseline vs After Remineralization)**				
- Min-Max	-30.09 - 19.97	-17.20 - 14.24	-37.01 - -16.49	F_(df=2)_=16.412 *p*<.001*
- Mean±SD	-5.61^**a,b**^ ±12.43	-2.56^**a,b**^±9.83	-26.24^**c**^±7.21	
- SE of Mean	3.93	3.11	2.28	
- 95.0% CI for Mean	-14.50 - 3.29	-9.59 - 4.47	-31.39 - -21.08	
- 25^th^ Percentile – 75^th^ Percentile	-10.20 - -1.84	-8.99 - 3.16	-31.94 - -17.56	
**Percentage Change (%) (After Demineralization vs After Remineralization)**				
- Min-Max	-32.42 - 66.41	23.36 - 61.84	1.73 - 60.86	F_(df=2)_=4.686 *p*=.018*
- Mean±SD	34.20^**a,b,c**^ ±27.93	40.88^**a,b**^±12.23	13.94^**a,c**^±18.18	
- SE of Mean	8.83	3.87	5.75	
- 95.0% CI for Mean	14.21 - 54.18	32.13 - 49.63	0.93 - 26.94	
- 25^th^ Percentile – 75^th^ Percentile	27.05 - 49.95	32.54 - 51.11	4.71 - 14.77	

Bonferroni Pairwise multiple comparison: mean of groups that is labeled with similar superscript letter are statistically not significantly different

*Min. – Max*. Minimum – Maximum, *S.D.* Standard Deviation, *SE* Standard error, CI: Confidence interval, *df* degree of freedom, *GG* Greenhouse-Geisser correction

^*^: Statistically significant (*p*<.05), NS: Statistically not significant (*p*≥.05)

Superscript letters for intergroup comparison: Biomin group assigned letter (a), Biomin and laser group assigned letter (b), and control group assigned letter (c)

Superscript letters for intragroup comparison: Baseline assigned letter (x), After Demineralization assigned letter (y), and after Remineralization assigned letter (z)

**Table 4:** Comparison of Phosphorus level (% mass) at different repeated points of the measurement interval in the three studied groups

**P (% mass)**	**Progression**			**Testof significance** ***p* value**
	**Biomin** **(***n***=10)**	**Biomin and Laser** **(***n***=10)**	**Control** **(***n***=10)**	
**Baseline**				
- Min-Max	14.93-17.32	14.29-17.23	14.00-17.62	F_(df=2)_=0.752 *p*=.529 NS
- Mean±SD	16.38^**X,Z**^±0.84	15.90^**X,Z**^±0.88	16.21^**X**^±0.96	
- SE of Mean	0.27	0.28	0.30	
- 95.0% CI for Mean	15.78-16.98	15.27-16.52	15.52-16.90	
- 25^th^ Percentile – 75^th^ Percentile	16.04-17.01	15.75-16.39	16.10-16.59	
**After Demineralization**				
- Min-Max	9.40-12.78	8.14-13.48	8.28-12.56	F_(df=2)_=0.555 *p*=.581 NS
- Mean±SD	10.77^**Y**^±0.96	11.34^**Y**^±1.86	10.76^**Y**^±1.27	
- SE of Mean	0.30	0.59	0.40	
- 95.0% CI for Mean	10.09-11.46	10.02-12.67	9.85-11.67	
- 25^th^ Percentile – 75^th^ Percentile	10.35-10.96	9.23-12.50	10.29-11.76	
**After Remineralization**				
- Min-Max	15.47-18.38	13.06-17.11	12.30-15.68	F_(df=2)_=16.056 *p*<.001*
- Mean±SD	16.72^**a,b,X,Z**^±0.82	16.01^**a,b,X,Z**^±1.23	14.04^**c,Z**^±1.18	
- SE of Mean	0.26	0.39	0.37	
- 95.0% CI for Mean	16.13-17.31	15.13-16.90	13.20-14.89	
- 25^th^ Percentile – 75^th^ Percentile	16.29-16.94	15.44-16.89	13.44-15.01	
**One-way repeated measures analysis** *p* value	F_(df=2)_=140.679 *p*<.001*	F_(df=2)_=39.902 *p*<.001*	F_(df=2)_=50.919 *p*<.001*	
**Percentage Change (%) (Baseline vs After Demineralization)**				
- Min-Max	-45.73 - -23.06	-49.19 - -15.06	-49.05 - -18.79	F_(df=2)_=0.919 *p*=.411 NS
- Mean±SD	-34.07±6.69	-28.27±13.24	-33.23±10.02	
- SE of Mean	2.12	4.19	3.17	
- 95.0% CI for Mean	-38.86 - -29.28	-37.74 - -18.80	-40.39 - -26.07	
- 25^th^ Percentile – 75^th^ Percentile	-38.86 - -28.86	-43.29 - -17.96	-37.67 - -24.42	
**Percentage Change (%) (Baseline vs After Remineralization)**				
- Min-Max	-4.25 - 14.59	-24.20 - 18.33	-24.47 - -2.93	F_(df=2)_=9.111 *p*=.001*
- Mean±SD	2.24^**a,b**^±5.81	1.26^**a,b**^±11.96	-13.15^**c**^±8.21	
- SE of Mean	1.84	3.78	2.60	
- 95.0% CI for Mean	-1.91-6.39	-7.30 - 9.82	-19.02 - -7.27	
- 25^th^ Percentile – 75^th^ Percentile	-1.99-3.34	-3.62 - 7.81	-20.89 - -4.89	
**Percentage Change (%) (After Demineralization vs After Remineralization)**				
- Min-Max	28.72 - 88.72	24.18-89.68	11.62-81.28	F_(df=2)_=3.259 *p*=.054 NS
- Mean±SD	56.52±18.02	43.88±21.26	32.60±23.26	
- SE of Mean	5.70	6.72	7.35	
- 95.0% CI for Mean	43.63-69.41	28.67-59.10	15.96-49.24	
- 25^th^ Percentile – 75^th^ Percentile	45.26-62.88	31.41-44.90	14.29-47.18	

Bonferroni Pairwise multiple comparison: mean of groups that is labeled with similar superscript letter are statistically not significantly different

*Min. – Max*. Minimum – Maximum, *S.D.* Standard Deviation, *SE* Standard error, CI: Confidence interval, *df* degree of freedom

^*^: Statistically significant (*p*<.05), NS: Statistically not significant (*p*≥.05)

Superscript letters: Biomin group assigned letter (a), Biomin and laser group assigned letter (b), and control group assigned letter (c)

Superscript letters for intragroup comparison: Baseline assigned letter (x), After Demineralization assigned letter (y), and after Remineralization assigned letter (z)

**Table 5:** Comparison of Fluoride level (% mass) at different repeated points of the measurement interval in the three studied groups

**F (% mass)**	**Progression**			**Testof significance** ***p* value**
	**Biomin** **(***n***=10)**	**Biomin and Laser** **(***n***=10)**	**Control** **(***n***=10)**	
**Baseline**				
- Min-Max	0.63 – 1.44	0.52 – 1.38	0.53 – 1.17	F_(df=2)_=1.098 *p*=.348 NS
- Mean±SD	0.94^**X,Z**^ ± 0.27	0.87^**X,Z**^± 0.28	0.78^**X**^ ± .20	
- SE of Mean	0.08	0.09	0.06	
- 95.0% CI for Mean	0.75 – 1.13	0.67 – 1.07	0.63 – 0.92	
- 25^th^ Percentile – 75^th^ Percentile	0.76 – 1.18	0.61 – 1.04	0.61 – 0.90	
**After Demineralization**				
- Min-Max	0.29 – 1.17	0.20 – 0.98	0.50 – 0.80	F_(df=2)_=0.037 *p*=.967 NS
- Mean±SD	0.62^**Y**^ ± .29	0.61^**Y**^ ± 0.29	0.64^**Y**^ ± 0.09	
- SE of Mean	0.09	0.09	0.03	
- 95.0% CI for Mean	0.42 – 0.83	0.41 – 0.82	0.58 – 0.70	
- 25^th^ Percentile – 75^th^ Percentile	0.37 – 0.83	0.35 – 0.82	0.61 – 0.68	
**After Remineralization**				
- Min-Max	0.39 – 1.40	0.45 – 1.50	0.35 – 0.84	F_(df=2)_=6.241 *p*=.006*
- Mean±SD	0.97^**a,b,X,Z**^± 0.29	0.98^**a,b,X,Z**^ ± 0.30	0.63^**c,Z**^ ± 0.14	
- SE of Mean	0.09	0.09	0.04	
- 95.0% CI for Mean	0.76 – 1.17	0.77 – 1.19	0.53 – 0.73	
- 25^th^ Percentile – 75^th^ Percentile	0.77 – 1.16	0.72 – 1.16	0.56 – 0.74	
**One-way repeated measures analysis** *p* value	F_(df=2)_=9.465 *p=*.002*	F_(df=2)_=17.450 *p*<.001*	F_(df=2)_=4.719 *p*=.023*	
**Percentage Change (%) (Baseline vs After Demineralization)**				
- Min-Max	-61.84 – 31.75	-65.57 – 27.63	-32.43 – 28.30	F_(df=2)_=1.985 *p*=.157 NS
- Mean±SD	-33.32 ± 27.54	-31.71 ± 25.42	-13.59 ± 20.26	
- SE of Mean	8.71	8.04	6.41	
- 95.0% CI for Mean	-53.02 – -13.62	-49.89 – -13.53	-28.08 – 0.90	
- 25^th^ Percentile – 75^th^ Percentile	-54.32 – -19.49	-41.57 – -28.70	-30.00 – 1.64	
**Percentage Change (%) (Baseline vs After Remineralization)**				
- Min-Max	-58.51 – 43.21	-26.23 – 97.37	-61.11 – 21.31	F_(df=2)_=2.892 *p*=.073 NS
- Mean±SD	5.53 ± 28.57	16.59 ± 35.21	-14.99 ± 24.63	
- SE of Mean	9.04	11.13	7.79	
- 95.0% CI for Mean	-14.91 – 25.97	-8.60 – 41.77	-32.61 – 2.63	
- 25^th^ Percentile – 75^th^ Percentile	-7.32 – 22.22	-6.96 – 32.69	-29.67 – 5.66	
**Percentage Change (%) (After Demineralization vs After Remineralization)**				
- Min-Max	-39.06 – 213.51	19.39 – 260.00	-44.44 - 19.35	F_(df=2)_=5.442 *p*=.010*
- Mean±SD	83.08^**a,b**^ ± 90.41	83.91^**a,b**^ ± 69.61	-1.79^**c**^ ± 18.65	
- SE of Mean	28.59	22.01	5.90	
- 95.0% CI for Mean	18.41 – 147.75	34.11 – 133.71	-15.13 – 11.54	
- 25^th^ Percentile – 75^th^ Percentile	9.47 – 185.37	35.80 – 97.14	-12.33 – 5.00	

Bonferroni Pairwise multiple comparison: mean of groups that is labeled with similar superscript letter are statistically not significantly different

*Min. – Max*. Minimum – Maximum, *S.D.* Standard Deviation, *SE* Standard error, CI: Confidence interval, *df* degree of freedom

^*^: Statistically significant (*p*<.05), NS: Statistically not significant (*p*≥.05)

Superscript letters for intergroup comparison: Biomin group assigned letter (a), Biomin and laser group assigned letter (b), and control group assigned letter (c)

Superscript letters for intragroup comparison: Baseline assigned letter (x), After Demineralization assigned letter (y), and after Remineralization assigned letter (z)

### 1) Mineral content (Ca,P,F) using EDX:

- Comparison between baseline and (After demineralization)

At baseline, there was no statistical significant difference between groups A,B and C regarding to mass% value of Ca, P and F.

**Fig. 7:**
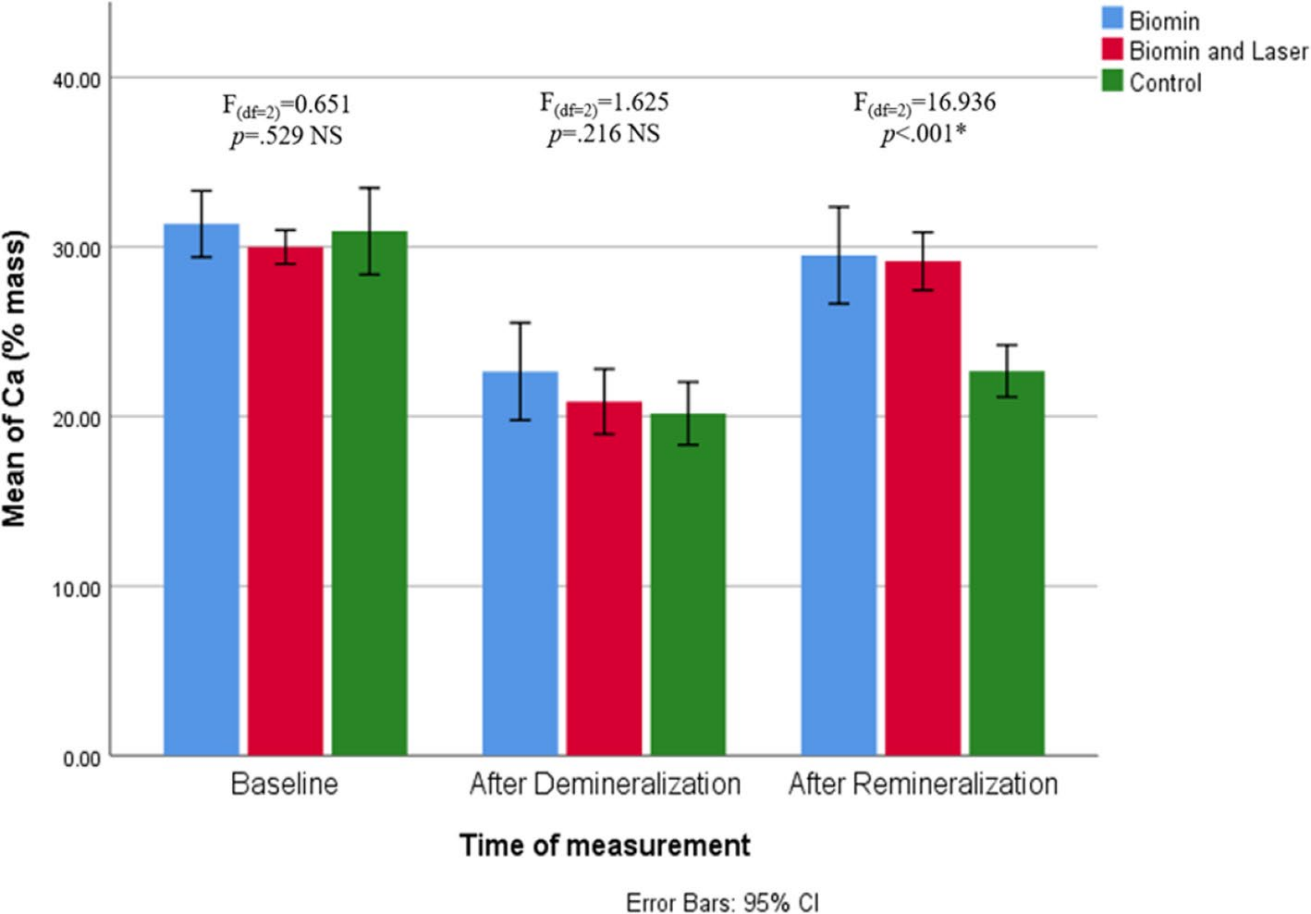
Clustered bar chart of Mean of Ca (% mass) in the studied groups at different times of measurement

After demineralization, it was shown that calcium level (% mass) significantly decreased in the three studied groups .Calcium level decreased from a mean of 31.34 ,29.98 and 30.91 (%mass) in groups A,B and C respectively at baseline to a mean of 22.64 ,20.86 and 20.16 % mass after demineralization (Fig. 7 and Table 3).

**Fig. 8:**
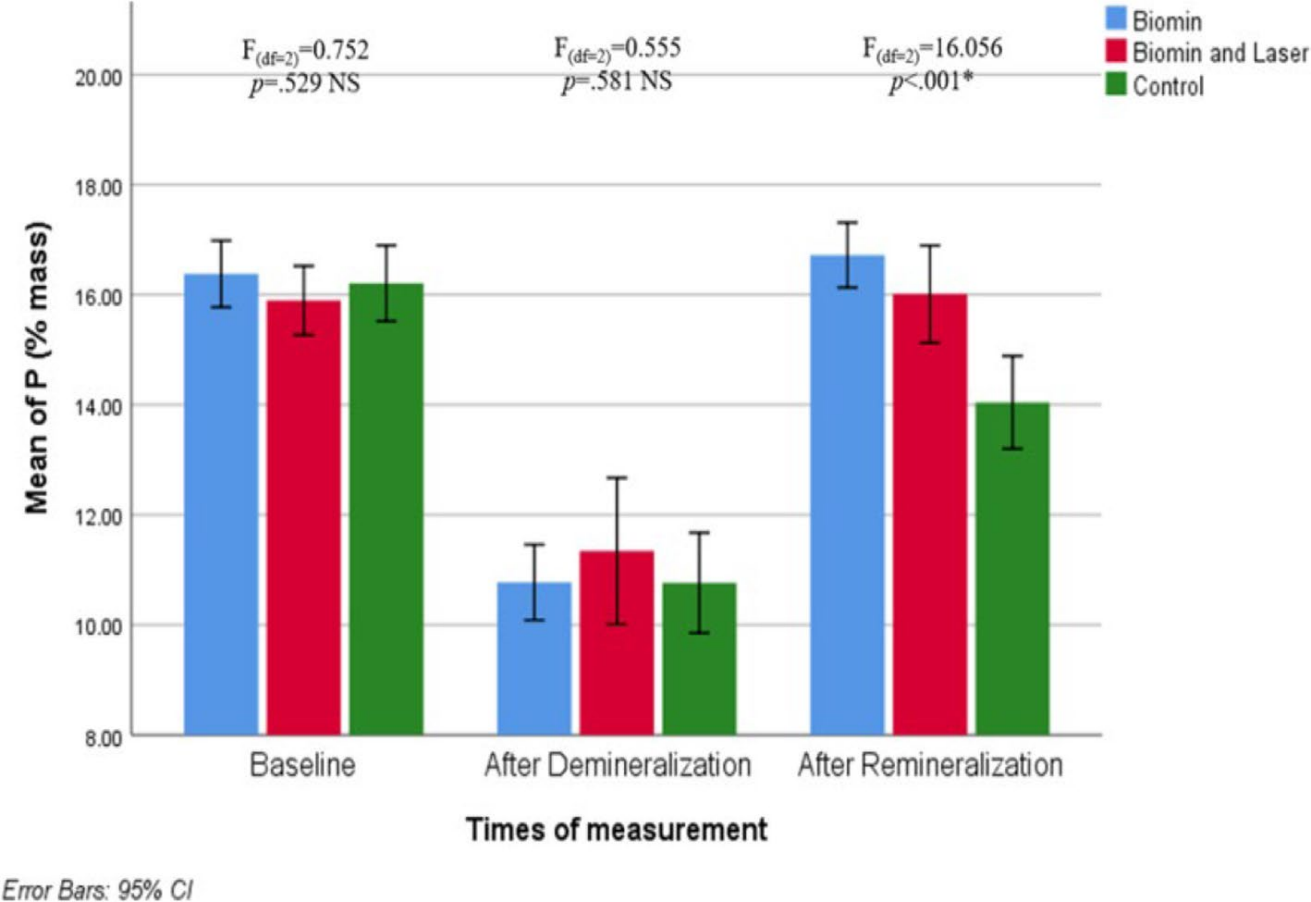
Clustered bar chart of Mean of P (% mass) in the studied groups at different times of measurement

Phosphorus level % mass also significantly decreased from a mean of 16.38, 15.90 and 16.21 to 10.77, 11.34 and 10.76 %mass in groups A,B and C respectively (Fig. 8 and Table 4).

**Fig. 9:**
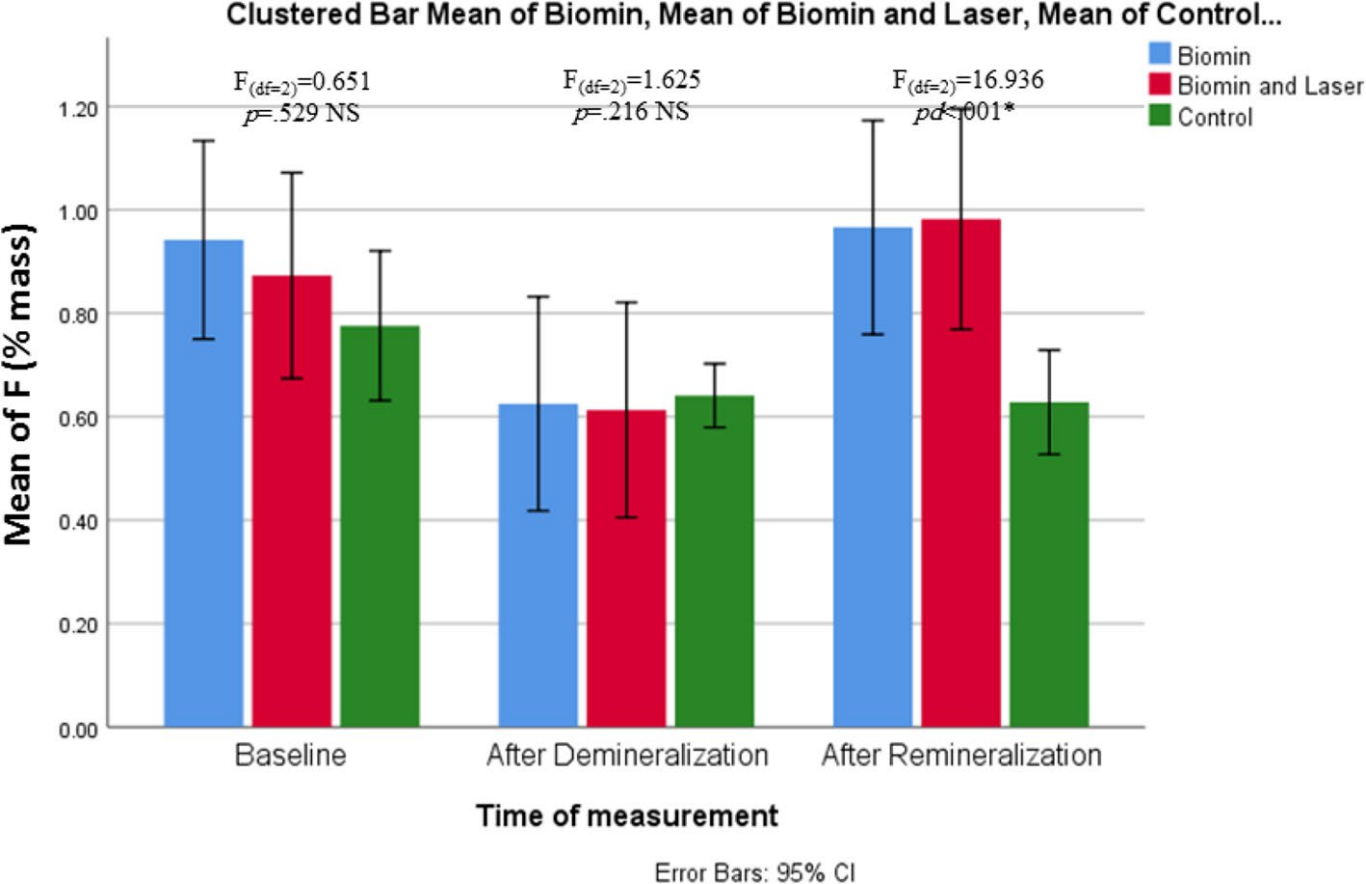
Clustered bar chart of Mean of F (% mass) in the studied groups at different times of measurement

Regarding to fluoride level (%mass), there was significant decrease from 0.94, 0.87 and 0.78 to 0.62, 0.61 and 0.64 in groups A,B and C respectively (*p*<0.05) (Fig. 9 and Table 5).

There was no statistical significant difference among the three studied groups in Ca,P,F level after demineralization (*p*≥0.05).

b. Comparison between (after demineralization) and (after remineralization):

After the application of the remineralizing agents, calcium ions gained a significant increase in groups A and B. Group B showed the highest gain of ions as percentage change (%) was 40.88±12.23. There was no statistical difference between group A and B after remineralization (*p*≥0.05) and both were significantly higher than the control group (*p<*0.05) (Table 3 and Fig. 7).

As regards the phosphorus ions, a significant increase was observed in all groups. Group A showed the highest gain as P level percentage change(%mass) was 56.52±18.02 and group C showed the least gain as P level percentage change was 32.60±23.26 .There was no significant difference between group A and group B but both were significantly higher than group C after remineralization (*p*<0.001) (Fig. 8 and Table 4).

Fluoride ions increased significantly in groups A and B (*p<*0.05) but decreased significantly in group C as shown in percentage change formula (-1.78±18.65). There was no statistical difference between group A and B (*p*≥.05) in fluoride level after remineralization (Fig. 9 and Table 5). Both groups A and B were significantly higher than group C (*p*=.006).

c. Comparison between baseline and after remineralization

When comparing the mean values of minerals % mass between the baseline and the last stage after application of the remineralizing agents, it was obvious that there was no significant difference in minerals percentage (Ca,P,F) between baseline and after remineralization in groups A and B (*p*≥0.05).

While in the control group, there was significant decrease in the Ca,P and F %mass between baseline and after remineralization process.

### 2. Environmental scanning *electron* microscope (ESEM) analysis

**Fig. 10:**
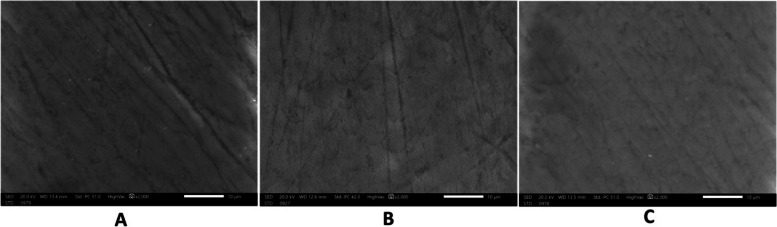
At the base line, micrographs (**a**) sample 9 in group a (**b**) sample 6 in group b (**c**) sample 5 in group c showed sound enamel with smooth surface .The enamel prisms showed minimal visibility, and the presence of scratches suggests potential carbon surface contamination

**Fig. 11:**
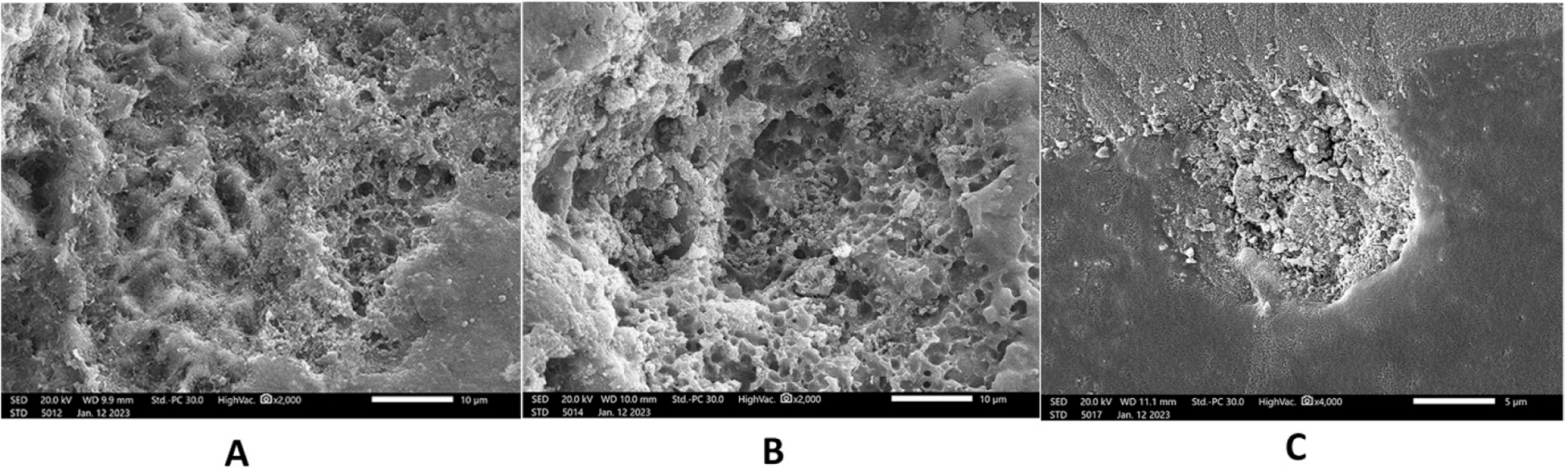
After demineralization ,micrographs (**a**) sample 3 in group a (**b**) sample 14 in group b (**c**) sample 11 in group c showed areas of dissolution and pores characterized by honey comb appearance. Enamel prisms become more visible because of erosion

**Fig. 12:**
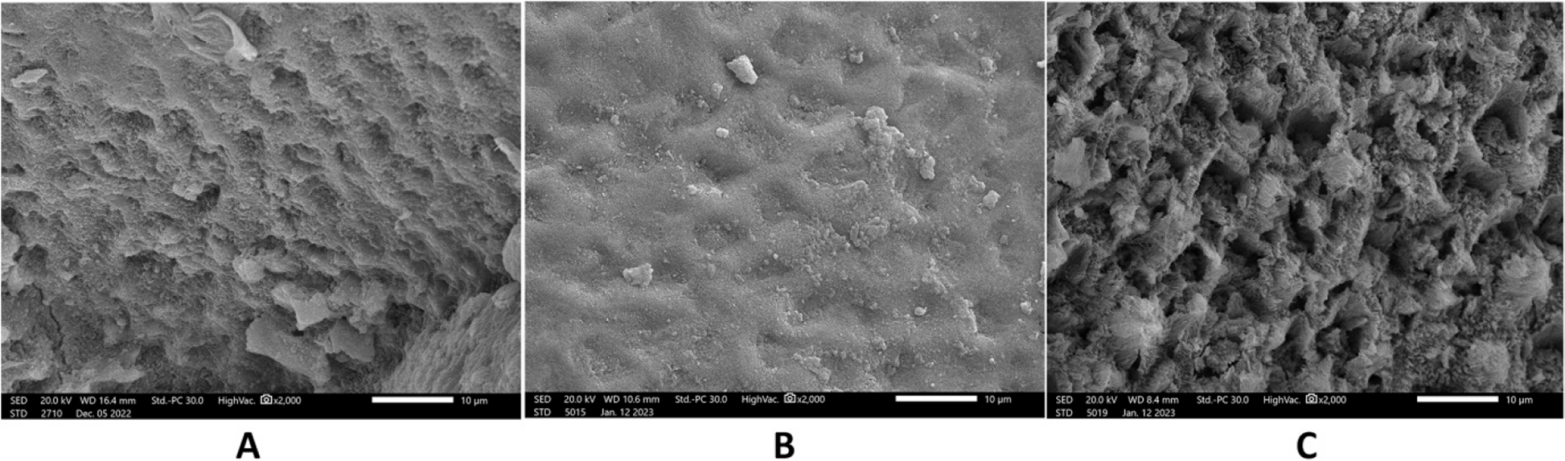
After remineralization ,biomin group showed enamel surface with partial restoration of the surface structure and areas of uniform and smooth enamel surface. Mineral crystals were deposited obliterating prism cores. **a** sample 22 **b** sample 27 **c** sample 28 in biomin group

**Fig. 13:**
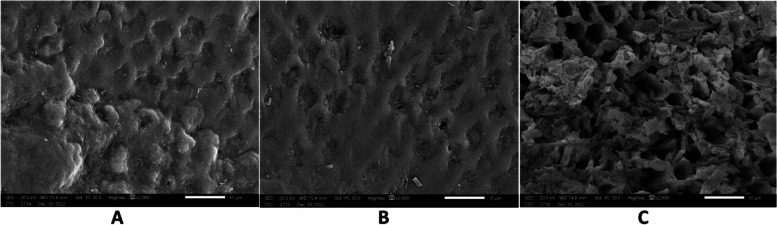
After remineralization in biomin and laser group micrographs showed areas of crystal deposition obliterating prism cores and smooth areas at the surface of enamel **a** sample 23 **b** sample 4 **c** sample 14 in biomin and laser group

**Fig. 14:**
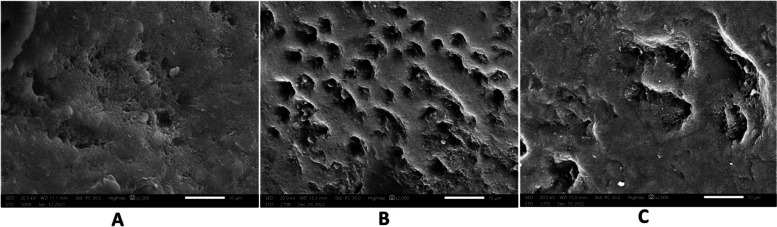
In the control goup, micrographs showed areas of erosions with visible prisms Sample 10 (**b**) sample 17 (**c**) sample 12 in the control group

The sample surface characteristics at each stage were described using ESEM at a magnification of x2000. At base line, micrographs showed smooth enamel surface. After demineralization, samples showed honey comb appearance which represent areas of minerals dissolution. After application of the remineralizing agent, samples showed partial restoration of enamel surface structure (Figs. 10, 11, 12, 13 and 14).

## Discussion

This study was conducted to evaluate the impact of diode laser and Biomin F toothpaste on the remineralization of WSLs.

The null hypothesis was accepted as there was no significant difference between the effect of Biomin F toothpaste coupled with diode laser and Biomin F alone on the remineralization of white spot lesions.

The natural physiological processes of demineralization and remineralization in tooth structure may be disrupted when there is an imbalance between pathogenic and protective factors [16, 18]. From a scientific standpoint, it has been recognized that salivary dysfunction, fermentable carbohydrates, and cariogenic bacteria play significant roles as pathogenic factors [26]. Calcium and phosphate ions presence in a supersaturated state within human saliva enables it to potentially facilitate the remineralization of enamel [38]. However, if acid challenges exceed this physiological remineralization process, alternative therapeutic interventions are needed to strengthen remineralization [17, 26].

Therefore, Biomin F was utilized in this study as it is a bioactive glass material containing a low level of fluoride. Ali et al [39] noted in their study that Biomin F toothpastes exhibited lower total fluoride content compared to the values asserted by their respective companies (approximately 400 ppm).

The majority of studies conducted on Biomin F have explored its efficacy in occluding dentinal tubules and addressing hypersensitivity [40, 41]. A systematic review focused on investigating the role of bioactive glass in enamel remineralization highlighted the significant contribution of fluoride-containing bioactive glass dentifrice, specifically Biomin F, in enamel regeneration [18].

Aidaros et al [15] carried out an in-vitro investigation utilizing SEM and elemental analysis to assess the mineral composition of extracted permanent third molars prior to and following the application of remineralizing agents, which included Biomin F. Their study involved comparing these agents, employing a similar application regimen to that of the current study (two minutes, twice daily for two weeks). They utilized the materials in the form of toothpaste and concluded that the combination of fluoride with bioactive glass technology, as seen in Biomin F toothpaste, had the most significant impact on the demineralized enamel surface. This finding aligned with our study, which demonstrated that Biomin F possessed the ability to remineralize white spot lesions (WSLs) to restore them to their baseline mineral content.

In this current study, laser was examined in conjunction with Biomin F, given that numerous studies have evaluated various types of lasers, including CO2, Nd:YAG, Er:YAG [42], and diode lasers:, utilizing different parameters for caries prevention and enamel remineralization, either with [43, 44] or without [45] fluoride-containing agents.

The present study revealed that the mineral content following remineralization reached the baseline mineral level in both test groups. This finding contradicted the results reported by Omran et al. [21] as the mean calcium mass percentage after remineralization was significantly lower compared to the baseline calcium mass percentage in the Biomin group. Notably, both studies employed the same Biomin F application protocol (two minutes twice daily for two weeks). This variation might be explained by the fact that Omran-T applied Biomin F as toothpaste slurries and subjected the samples to a shorter demineralization period (72 hours), while the present study utilized it in the form of toothpaste, as commonly used by orthodontic patients. Nonetheless, both studies concurred on the high phosphate content of Biomin F.

An intriguing observation emerged from the study comparing laser treatment with a bioactive glass material (Novamin) using SEM: laser therapy did not provide additional benefits to Novamin in the process of remineralizing the enamel surface [26]. In the current study, where Biomin F demonstrated the capability to remineralize the enamel surface, the diode laser did not exhibit a synergistic effect in enamel remineralization, as indicated by the insignificant difference between group A and B. Similarly, comparable outcomes were observed when Novamin was utilized alongside laser therapy.

The potential rationale for the findings of this study could be that the efficacy of the bioactive glass material (Biomin F) relies on its interaction with physiological aqueous solutions, leading to the release of calcium, phosphorus, and fluoride. However, when a diode laser is employed, a certain degree of heat is generated within the treated surface, typically ranging from 1 to 6 degrees Celsius [46]. As a consequence, this leads to some dryness and removal of moisture from the paste, which is essential for mineral release.

The analysis of SEM micrographs enabled us to observe the notable regeneration of the enamel structure and the deposition of mineral crystals, a result that aligns with findings reported by Bakrey et al., who observed the deposition of mineral crystals blocking the dentinal tubules [40] after using Biomin F.

This study has a limitation that we must consider the dynamic complex system in oral environment which may differ from the in-vitro study employed in the present work.

## Conclusion

Within the limitation of the present study, we concluded that Biomin F toothpaste is promising in repairing the white spot lesions on the surface of the demineralized enamel. Diode laser did not affect the remineralizing ability of Biomin F toothpaste.

## Recommendation

Clinical studies are needed for more evaluation of the benefits of these approaches. It is recommended to evaluate the synergistic effect between laser and biomin F in decreasing the white spot lesions depth with a variation of laser protocols and exposure time. Microhadness testing is recommended to confirm the remineralization effect.

## Data Availability

The data and materials used to support the findings of this study are available from the corresponding authors upon request.
